# Effects of a Web-Based Intervention on Physical Activity and Metabolism in Older Adults: Randomized Controlled Trial

**DOI:** 10.2196/jmir.2843

**Published:** 2013-11-06

**Authors:** Carolien A Wijsman, Rudi GJ Westendorp, Evert ALM Verhagen, Michael Catt, P Eline Slagboom, Anton JM de Craen, Karen Broekhuizen, Willem van Mechelen, Diana van Heemst, Frans van der Ouderaa, Simon P Mooijaart

**Affiliations:** ^1^Department of Gerontology and GeriatricsLeiden University Medical CenterLeidenNetherlands; ^2^Leyden Academy of Vitality and AgeingLeidenNetherlands; ^3^Department of Public and Occupational Health, EMGO+InstituteVU University Medical CenterAmsterdamNetherlands; ^4^Institute for Ageing and HealthNewcastle UniversityNewcastle upon TyneUnited Kingdom; ^5^Department of Medical StatisticsMolecular Epidemiology SectionLeiden University Medical CenterLeidenNetherlands; ^6^Netherlands Consortium for Healthy AgeingLeidenNetherlands; ^7^Institute for Evidence-Based Medicine in Old Age, IEMOLeidenNetherlands

**Keywords:** physical activity, Internet, accelerometry, aging, metabolism, self-monitoring

## Abstract

**Background:**

Lack of physical activity leads to detrimental changes in body composition and metabolism, functional decline, and increased risk of disease in old age. The potential of Web-assisted interventions for increasing physical activity and improving metabolism in older individuals holds great promise but to our knowledge it has not been studied.

**Objective:**

The goal of our study was to assess whether a Web-based intervention increases physical activity and improves metabolic health in inactive older adults.

**Methods:**

We conducted a 3-month randomized, waitlist-controlled trial in a volunteer sample of 235 inactive adults aged 60-70 years without diabetes. The intervention group received the Internet program Philips DirectLife, which was directed at increasing physical activity using monitoring and feedback by accelerometer and digital coaching. The primary outcome was relative increase in physical activity measured objectively using ankle- and wrist-worn accelerometers. Secondary outcomes of metabolic health included anthropometric measures and parameters of glucose metabolism.

**Results:**

In total, 226 participants (97%) completed the study. At the ankle, activity counts increased by 46% (standard error [SE] 7%) in the intervention group, compared to 12% (SE 3%) in the control group (*P*_difference_<.001). Measured at the wrist, activity counts increased by 11% (SE 3%) in the intervention group and 5% (SE 2%) in the control group (*P*_difference_=.11). After processing of the data, this corresponded to a daily increase of 11 minutes in moderate-to-vigorous activity in the intervention group versus 0 minutes in the control group (*P*_difference_=.001). Weight decreased significantly more in the intervention group compared to controls (−1.5 kg vs −0.8 kg respectively, *P*=.046), as did waist circumference (−2.3 cm vs −1.3 cm respectively, *P*=.036) and fat mass (−0.6% vs 0.07% respectively, *P*=.025). Furthermore, insulin and HbA1c levels were significantly more reduced in the intervention group compared to controls (both *P*<.05).

**Conclusions:**

This was the first study to show that in inactive older adults, a 3-month Web-based physical activity intervention was effective in increasing objectively measured daily physical activity and improving metabolic health. Such Web-based interventions provide novel opportunities for large scale prevention of metabolic deregulation in our rapidly aging population.

**Trial Registration:**

Dutch Trial Registry: NTR 3045; http://www.trialregister.nl/trialreg/admin/rctview.asp?TC=3045 (Archived by WebCite at http://www.webcitation.org/6KPw52dCc).

## Introduction

Lack of physical activity is perhaps one of the greatest risk factors for contemporary societal health problems. Insufficient physical activity contributes to obesity and has been associated with increased risks of cardiovascular disease [1,2], diabetes mellitus [3], cognitive decline [4,5], and premature mortality [6,7]. For the older age groups, this is of particular relevance as physical activity decreases with age, while the prevalence of metabolic disease and its complications increase as a function of age. Intervention studies directed at increasing physical activity have been shown to be effective and may improve metabolism [8], including the older populations [9,10]. However, most of these interventions have used face-to-face communication, making them costly and time-consuming, thus hampering the potential of implementation as preventive programs at a larger scale.

Modern technologies, such as Internet and email, provide interactive ways to administer digital coaching and feedback on physical activity and have potential for wide-scaled implementation. A recent meta-analysis on Web-based physical activity intervention studies showed promising results in increasing daily physical activity [11]. Many of these, however, used either small study populations, included study populations under the age of 60, or did not measure physical activity objectively. Moreover, it is unclear what the effect is of such Web-based physical activity interventions on outcomes of metabolic health in old age [12,13].

Since 2005, Internet use has doubled in 65-75 year olds in the Netherlands [14]; 70% of this age group is familiar with using the Internet and therefore provides a target for Web-based interventions. In this randomized controlled trial, we examined whether a 3-month Web-assisted intervention directed at increasing daily physical activity was effective in 60-70 year old inactive individuals. The intervention comprised an Internet program aimed at increasing physical activity, which focused on effective components of health behavior change including self-monitoring by accelerometer and goal setting with the help of digital coaching. Furthermore, we studied the effect of this intervention on metabolic health including anthropometric measures and markers of glucose and lipid metabolism.

## Methods

### Study Design and Participants

The study recruited participants aged 60-70 years from the region of Leiden, Netherlands. The recruitment strategy included advertisements in local newspapers and press notification, directing participants motivated to increase physical activity to the study website where they completed an online questionnaire that assessed the following inclusion and exclusion criteria: (1) age between 60 and 70 years, (2) no history of diabetes or use of glucose lowering medication, (3) absence of disability impeding increase in physical activity, and (4) possession and use of personal computer with Internet connection. If all of the above criteria were met, potential participants filled out an email address where they subsequently received a questionnaire asking for current physical activity and personal data such as full name and address. The presence of an inactive lifestyle was then assessed by a self-reported physical activity questionnaire: general practice physical activity questionnaire, GPPAQ [15]. This yielded four categories of physical activity: inactive, moderately inactive, moderately active, and active. We defined inactive as having less than 3 hours of exercise and cycling combined weekly, corresponding to the inactive, moderately inactive, or moderately active category. Participants in the active category of GPPAQ did not meet inclusion criteria for our definition of an inactive lifestyle.

Subjects who met all inclusion criteria received detailed study information in print. If willing to participate, participants received an online questionnaire on education, smoking status, medical history, and medication use, and visited the study center at baseline and after 3 months (13 weeks). At the baseline visit, subjects were randomly assigned to the intervention group or a waitlist control group (in which the participants received the intervention program after 3 months when the study ended) by the study physician or research nurse. Randomization was performed by a computerized program for intervention versus waitlist control in a ratio of 1:1, with a block size of 12. Stratification was performed by gender. Concealment of treatment allocation was ensured by randomizing at the end of the first study visit, after all baseline measurements and instructions at the study center were completed. Blinding of this intervention was not possible and therefore not applied. Written informed consent was obtained from all subjects. The study was approved by the medical ethical committee of Leiden University Medical Center, Netherlands. An independent physician was available for questions regarding study information.

### Sample Size Calculation

The sample size of the study was based on the assumption of a mean 10% higher increase (SD 25%) in daily physical activity counts as measured using accelerometers on the ankle and wrist in the intervention group compared to the control group during a 3-month period. For this effect size with a power of 0.80 at alpha 0.05 (two-sided), we calculated a sample size of 198 participants for the intention-to-treat (ITT) analysis. Based on an estimated drop-out rate of 15%, we aimed to include 232 participants and stopped after successful inclusion of 235 participants.

### Intervention

Subjects in the intervention group received a commercially available Web-based physical activity program (DirectLife, Philips, Consumer Lifestyle, Amsterdam) directed at increasing daily physical activity. The DirectLife program is based on the stages of change and I-change health behavior change models [16,17] and takes into account the individual’s current activity level. It then provides a personal goal. Briefly, DirectLife consists of three elements: (1) an accelerometer-based activity monitor, (2) a personal website, and (3) a personal e-coach, who provides regular updates of the individual’s physical activity status by email and gives advice to increase physical activities (Multimedia Appendix 1). By means of these elements, the program aims to increase awareness about one’s own physical activity behavior, to give feedback on recent actual physical activity, and to provide support to make sustainable changes in physical activity behavior. The activity monitor of DirectLife is based on the Tracmor tri-axial accelerometer and has been validated against doubly labeled water for the estimation of total daily life energy expenditure [18]. The DirectLife monitor is the consumer version of the Tracmor. Intervention group participants received the program, including the accelerometer, directly after randomization at the first study visit. They then received a link by email for registration and access to the Web program. Participants of the program were instructed to continuously wear the activity monitor throughout the day to measure daily physical activity (Multimedia Appendix 2). Data were uploaded through an Internet connection to the database of the commercial provider. After an initial 8-day “assessment period” starting 1 week after the study visit, in which the current level of daily activity was measured, a target was set to increase the level of daily activity during a 12-week Web-based interactive coaching program. Participants were given a target for daily activity, which increased weekly, and data from the accelerometer were used for regular feedback (Multimedia Appendix 3). Coaching included general recommendations on physical activities, and coaches were available for further questions and advice by email correspondence. All participants were in contact with one of the digital coaches available for the DirectLife program during the entire study period.

The control group was placed on a 3-month waiting list, after which they received access to the intervention program at the end of the study. No specific instructions regarding daily physical activity were given.

### Measurements

#### Baseline Questionnaire

Enrollment and follow-up took place from November 2011 to August 2012. In preparation for the first visit to the study center, all participants completed a Web-delivered questionnaire on education, smoking status, and medical history, including medication use. Education was categorized as low (primary education and lower vocational education), intermediate (secondary education and intermediate vocational education), or high (high vocational education and university).

#### Primary Outcome

At baseline and 3-month follow-up, daily physical activity was measured during 7 days following the visit at the study center, using an ankle- and wrist-worn tri-axial accelerometer (GENEActiv, Kimbolton, Cambs, United Kingdom). Wear was started on a random weekday, and GENEActive monitors were returned after 7 days by standard mail. We chose to assess the primary outcome using accelerometers other than the one included in the intervention program in order to avoid interpretation of the intervention as an outcome. Both GENEActiv monitors were worn 24 hours per day on the right side. The GENEActiv wrist accelerometer provides a simple summary statistic of total activity counts has been validated for measuring daily physical activity against doubly labeled water [19]. We chose to additionally assess total activity counts using an ankle accelerometer as we hypothesized that this location would be more sensitive to walking and cycling behavior [20,21], the latter being a very frequent activity in our target population in the Netherlands. Primary outcome was the individual’s relative change in activity counts after the intervention compared to baseline, measured at wrist and ankle. As a derivative outcome, we calculated from the wrist accelerometer the minutes per day spent in moderate or vigorous activity, which has been validated against indirect calorimetry [22].

Measurement frequency was set at 85.7 Hz, and raw acceleration values in “g” were recorded continuously on each axis over 7 consecutive days. Prior to processing, data were plotted for visual identification of non-wear and device faults. Non-wear was determined visually using thresholds of movement in combination with self-reported non-wear from participants. Short periods of non-wear (eg, bathing) were accepted, and data for these periods were not imputed. Accelerometer data from participants contributing to GENEActiv data for 5 days or more within the 7-day period were included in the analysis. Data from each axis were processed by a high pass RC filter (fc=0.27 HZ) before computation of the resultant acceleration for each recorded time point: R=(x^2^ + y^2^ + z^2^)^0.5^. The average of each 24-hour integral of these values over the first 5 days was used as the average daily activity count for the assessment period. As a measure of physical activity, we used total activity counts recorded at both the ankle and the wrist accelerometer independently. Data collected from the right wrist of each participant over 5 days of continuous movement monitoring were processed to yield activity counts for successive 1-minute epochs and classified according to the appropriate MET cut-off points according to the method of Esliger [22], to establish the average number of minutes (epochs) spent daily in moderate and vigorous physical activity. Outcome assessment was done by an independent researcher who was blind to study arm allocation (MC).

#### Secondary Outcomes

Body height was measured without shoes using a stadiometer. Body weight was assessed at both visits without shoes using a measurement scale. Waist circumference was obtained in a standing position halfway between the anterior superior iliac spine and the lower rib. Hip circumference was measured halfway between the trochanter major and the iliac crest.

Lean body mass and body fat percentage were assessed by bio-electrical impedance (BIA) analysis using a commercial portable device with hand-to-foot single frequency measurement (Biostat 1500, Euromedix, Leuven, Belgium). Blood pressure was measured twice at each visit using a hand-held sphygmomanometer after 5 minutes of lying down. The mean of the two consecutive measurements was used. Heart rate was measured at the wrist after at least 5 minutes of lying down. Grip strength was measured to the nearest kilogram three times using a Jamar hand dynamometer (Sammons Preston, Inc, Bolingbrook, IL, United States) with the dominant hand. The highest value was used for analysis. Framingham risk scores were calculated using NIH criteria [23].

#### Biochemical Assessments

Fasting blood samples were drawn from each participant at both visits in the morning. Samples were transferred to the lab within 2 hours, aliquotted, and frozen at −80°C. All serum measurements were performed in one batch after completion of the entire study with fully automated equipment. Fasting glucose, cholesterol, HDL-cholesterol, and triglyceride levels were determined using the Modular P2 analyzer (Roche, Almere, Netherlands), and fasting serum insulin using immunoassay by Immulite 2500 (DPC, Los Angeles, CA, United States). Glycated hemoglobin was determined by high-performance liquid chromatography (Primus Ultra2, Trinity Biotech Company, Kansas City, MO, United States). C-reactive protein (hsCRP) was determined using a high-sensitive immunoassay (COBAS integra, Roche, IN, United States). Low density lipoprotein (LDL) cholesterol was calculated using the Friedewald formula in participants without hypertriglyceridemia [24].

#### Statistical Analyses

Differences between baseline and follow-up within groups were tested using a paired sample Student *t* test of the means. For skewed variables, ln (natural logarithm) transformation was used. The effect of the intervention on physical activity was assessed by an unpaired 2-sided *t* test, comparing the relative change in daily physical activity counts between the intervention group and control group. For relative change in moderate-to-vigorous physical activity, a nonparametric test was used due to skewness of data. The effect of the intervention on secondary outcomes was assessed using an unpaired 2-sided *t* test, comparing the change in the secondary outcome between the intervention group and control group. Primary analyses were performed by ITT principle. Our study did have one follow-up measurement only, and loss to follow-up was very low. We therefore did not use imputation to replace our data, and participants from whom data were lost were not in the ITT analysis. For per-protocol analysis, we included in the intervention group only those participants who finished the 12-week plan of the intervention program, as assessed by uploaded accelerometer data of the participant in week 12 of the DirectLife intervention program. All analyses were performed with SPSS version 20.0. Statistical significance was accepted at *P*<.05.

## Results

### Participant Characteristics

**Figure 1:**
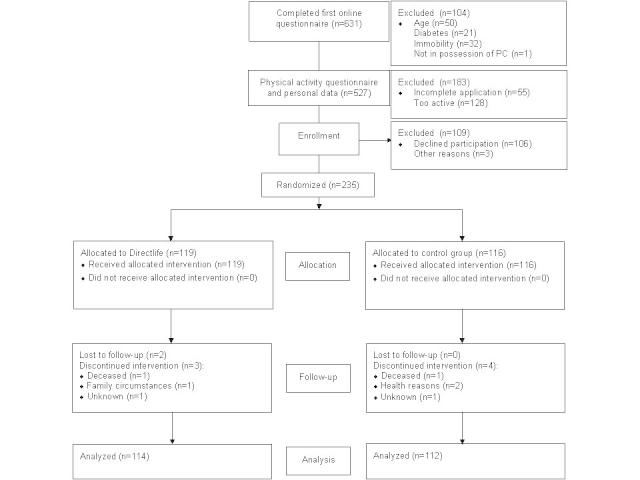
Consort flowchart of participants.

Figure 1 shows the inclusion flow chart of participants. A total of 631 participants completed the questionnaire on the study website. Of those, 235 participants (37%) met inclusion criteria and were randomized: 119 in the intervention arm, 116 in the control arm. Nine participants did not complete the study: 5 and 4 participants in the intervention and control group, respectively. Final analyses of outcomes therefore included 114 participants in the intervention group and 112 control participants.

Baseline characteristics of randomized participants are shown in Table 1. The study groups were similar for all parameters. The majority of participants were male and middle or highly educated. A substantial number of the study participants used antihypertensive medication: 46% and 38 % of participants in intervention and control group, respectively. On average, participants were overweight with a mean BMI of 28.9 kg/m^2^ (SD 4.7) and 29.l kg/m^2^ (SD 4.7) in the intervention and control group, respectively. There was no significant difference in baseline activity level between groups.

**Table 1:** Baseline characteristics of study participants (data are presented as medians with interquartile range [IQR] when skewed; alcohol use was calculated only in those who reported drinking alcohol [n=102 for intervention and n=101 for control]).

Characteristics		Intervention (n=119)	Control (n=116)
**Demographics**			
	Female sex, n (%)	47 (39.5)	49 (42.2)
	Age, yrs (mean, SD)	64.7 (3.0)	64.9 (2.8)
**Degree of self-reported activity, n (%)**			
	Moderately active	41 (34.5)	48 (41.4)
	Moderately inactive	36 (30.3)	34 (29.3)
	Inactive	42 (35.3)	34 (29.3)
**Level of education, n (%)**			
	Low	7 (5.9)	2 (1.7)
	Intermediate	45 (37.8)	46 (39.7)
	High	66 (55.5)	67 (57.8)
	Current smoking	7 (5.9)	8 (6.9)
	Alcohol use, units/wk (mean, SD)	12.0 (8.0)	10.2 (9.4)
**Medical history and medication use, n (%)**			
	Coronary heart disease^a^	14 (11.8)	16 (13.8)
	Arrhythmia	10 (8.4)	14 (12.1)
	Lung emphysema	3 (2.5)	5 (4.3)
	Stroke	6 (5.0)	3(2.6)
	Malignancies	17 (14.3)	17 (14.7)
	Thyroid disease	8 (6.7)	8 (6.9)
	Antihypertensive use	55 (46.2)	44 (37.9)
	Statin use	31 (26.1)	24 (20.7)
	Anticoagulant use	25 (21.0)	20 (17.2)
	Psychotropic use	11 (9.2)	16 (13.8)
**Physical activity**			
	Ankle monitor, counts/day (mean, SD)	3.68×10^5^ (1.72×10^5^)	3.78×10^5^ (1.84×10^5^)
	Wrist monitor, counts/day (mean, SD)	3.51×10^5^ (1.10×10^5^)	3.39×10^5^ (1.16×10^5^)
	MVPA^b^, min/day, median (IQR)	16.8 (7.8-26.4)	14.4 (8.2-32.0)
**Clinical parameters, mean (SD)**			
	Height (cm)	173.6 (9.9)	172.1 (9.3)
	Weight (kg)	87.4 (15.8)	86.3 (15.8)
	BMI^c^ (kg/m^2^)	28.9 (4.7)	29.1 (4.7)
	Waist circumference (cm)	102.3 (13.1)	101.4 (12.3)
	Hip circumference (cm)	109.1 (9.1)	108.7 (8.9)
	Waist/hip ratio	0.93 (0.08)	0.93 (0.08)
	Fat percentage (%)	36.5 (7.6)	36.4 (8.1)
	Systolic blood pressure (mmHg)	146 (18)	145 (17)
	Diastolic blood pressure (mmHg)	86 (9)	86 (11)
	Resting heart rate (beats/min)	72 (10)	71 (11)
	Grip strength (kg)	37.5 (10.2)	37.9 (10.4)
	Framingham 10-year CVD^d^ risk %	11.9 (7.2)	11.3 (7.5)
**Biochemistry, mean (SD)**			
	Fasting venous glucose (mmol/L)	5.7 (0.7)	5.7 (0.7)
	Fasting insulin (mU/L) (median, IQR)	11.5 (8.1–16.9)	10.8 (7.0–15.8)
	HbA1c (%)	5.4 (0.3)	5.4 (0.3)
	HOMA^e^ index (median, IQR)	2.8 (2.0–4.4)	2.6 (1.7–4.3)
	Total cholesterol (mmol/L)	5.7 (1.1)	5.8 (1.0)
	HDL^f^ cholesterol (mmol/L)	1.5 (0.5)	1.4 (0.4)
	Triglycerides (median, IQR), (mmol/L)	1.5 (1.1–2.0)	1.4 (1.1–2.0)
	LDL^g^ cholesterol (mmol/L)	3.6 (1.0)	3.6 (0.9)
	Total/HDL cholesterol ratio	4.2 (1.3)	4.3 (1.3)
	C-reactive protein (median, IQR), (mg/L)	1.6 (0.8–3.1)	1.4 (0.8–4.1)

^a^Coronary heart disease: myocardial infarction/ angina pectoris.

^b^MVPA=moderate-to-vigorous physical activity.

^c^BMI=body mass index.

^d^CVD=cardiovascular disease.

^e^HOMA=homeostatic model assessment.

^f^HDL=high density lipoprotein.

^g^LDL=low density lipoprotein.

### Adherence to the Intervention Program

All 114 participants who completed the study in the intervention arm received the intervention program during the visit to the study site at baseline. Of these, 109 participants (95.6%) started the intervention program after completing the first assessment week of the intervention program. In total, 104 participants (91.2%) completed the 12-week intervention program.

### Primary Outcome: Physical Activity

**Figure 2:**
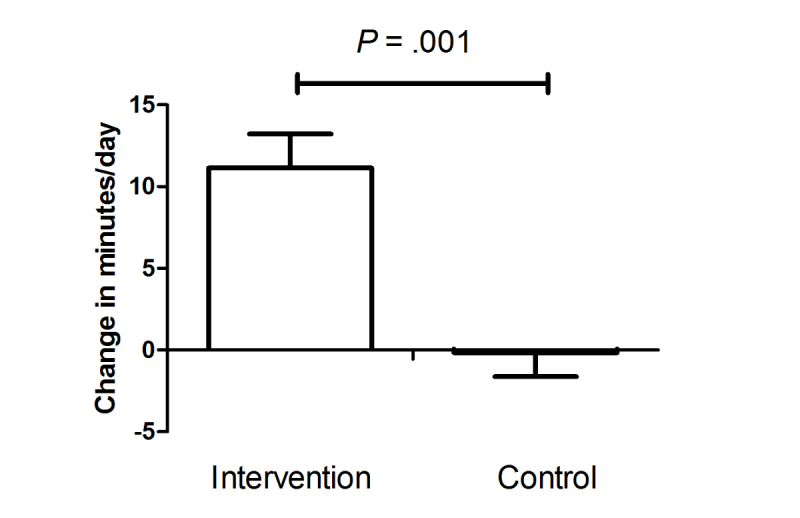
Change in daily physical activity expressed as moderate-to-vigorous physical activity measured at the wrist.

Accelerometer data were available for 107 intervention and 109 control participants for ankle monitors, and 108 and 105 intervention and control participants for wrist monitors, respectively. After 13 weeks, daily physical activity as measured by the ankle accelerometer increased by 46% (SE 7%, *P*<.001) in the intervention group, compared to 12 % (SE 3%, *P*<.001) in the control group (*P*_difference_<.001). Daily physical activity measured by the wrist accelerometer increased by 11% (SE 3%, *P*<.001) in the intervention group, and by 5% (SE 2%, *P*=.027) in the control group (*P*_difference_=.11). In the intervention group, there was a mean increase of 11.1 minutes per day (SE 2.1) spent in moderate-to-vigorous activity, compared to a mean decrease of 0.1 minutes (SE 1.5) in the control group (*P* for relative difference=.001) (Figure 2). In the per-protocol analysis, taking into account only those 91% (n=104) of participants who completed the intervention phase of the DirectLife program, results did not change.

### Secondary Outcomes: Parameters of Metabolic Health

**Figure 3:**
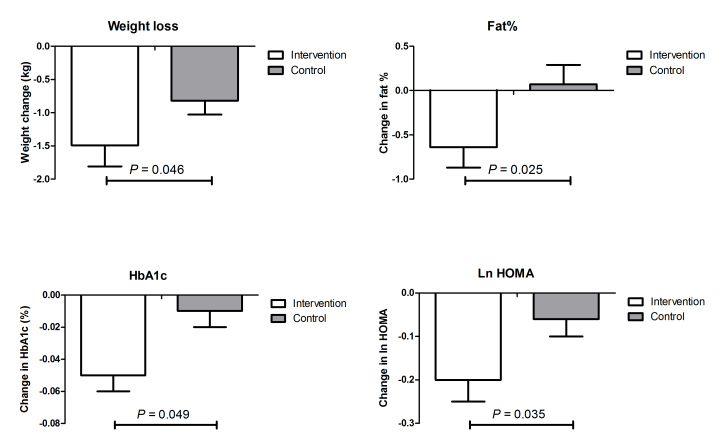
Changes in a selection of metabolic parameters.

Changes in parameters of metabolic health within and between groups are shown in Table 2 and Figure 3 (data represent mean changes with SE; *P* value for difference between intervention and control group using an unpaired 2-sided *t* test). A significant effect of the intervention on weight loss was seen with a mean change of −1.49 kg (SE 0.26) in the intervention group compared to −0.82 kg (SE 0.21) in the control group (*P*_difference_=.046). Likewise, waist circumference decreased more in the intervention vs control group: −2.33 SE 0.36 cm vs −1.29 SE 0.34 cm, *P*_difference_=.036. Fat percentage also decreased more in the intervention vs control group: −0.64 SE 0.23 vs 0.07 SE 0.22, *P*_difference_=.025. Glucose metabolism improved significantly; HbA1c decreased more in the intervention group (−0.05%, SE 0.01) compared to the control group (−0.01%, SE 0.01, *P*_difference_=.049). Similarly, fasting ln insulin levels (−0.16, SE 0.04 vs −0.04 SE 0.04, *P*_difference_=.037) improved significantly in the intervention group compared to controls, as did the HOMA-index (−0.20 SE 0.05 vs −0.06 SE 0.04, *P*_difference_=.035). Total cholesterol, LDL, and triglycerides levels improved in the intervention group, but these differences were not significant between groups. A decrease of more than 2 mmHg in systolic blood pressure was observed in both groups (*P*_difference_=.83) and a decrease in resting heart rate of over 5 beats per minute in the intervention group and almost 4 beats per minute in the control group (*P*_difference_=.15). No significant change in grip strength was seen in either of the groups. In the per-protocol analysis, taking into account only those 91% of participants who completed the intervention phase, differences did not materially change.

**Table 2:** Changes in parameters of metabolic health in study participants at follow-up (data represent mean differences with standard error [SE]).

		Intervention (n=114)		Control (n=112)		
		Mean difference (SE)	*P* value for change^a^	Mean difference (SE)	*P* value for change^a^	*P* value between groups^b^
**Clinical parameters**						
	Weight, kg	−1.49 (0.26)	<.001	−0.82 (0.21)	<.001	.046
	BMI^c^, kg/m^2^	−0.50 (0.09)	<.001	−0.29 (0.07)	<.001	.068
	Waist circumference, cm	−2.33 (0.36)	<.001	−1.29 (0.34)	<.001	.036
	Hip circumference, cm	−1.75 (0.33)	<.001	−1.12 (0.28)	<.001	.14
	Waist/hip ratio	−0.008 (0.004)	.032	−0.001 (0.003)	.81	.16
	Fat percentage, %	−0.64 (0.23)	.003	0.07 (0.22)	.91	.025
	Lean mass, kg	−0.37 ( 0.19)	.32	−0.57 (0.20)	.033	.46
	Systolic blood pressure (mmHg)	−2.73 (1.35)	.046	−2.30 (1.39)	.10	.83
	Diastolic blood pressure (mmHg)	1.10 (0.78)	.16	0.10 (0.82)	.91	.38
	Resting heart rate (beats/min)	−5.49 (0.94)	<.001	−3.68 (0.85)	<.001	.15
	Grip Strength (kg)	0.31 (0.38)	.41	0.00 (0.34)	1.00	.50
	Framingham 10-year risk %	−0.54 (0.33)	.11	−0.01 (0.31)	.98	.24
**Biochemistry**						
	Fasting glucose venous (mmol/L)	−0.20 (0.05)	<.001	−0.13 (0.04)	.001	.31
	Ln fasting insulin (mU/L)	−0.16 (0.04)	<.001	−0.04 (0.04)	.33	.037
	HbA1c (%)	−0.05 (0.01)	<.001	−0.01 (0.01)	.25	.049
	Ln HOMA^d^-index	−0.20 (0.05)	<.001	−0.06 (0.04)	.15	.035
	Total cholesterol (mmol/L)	−0.25 (0.06)	<.001	−0.18 (0.05)	.001	.36
	HDL^e^ cholesterol (mmol/L)	−0.008 ( 0.02)	.71	−0.04 (0.02)	.033	.27
	Ln Triglycerides (mmol/L)	−0.10 (0.03)	.003	−0.06 (0.02)	.023	.30
	LDL^f^ cholesterol (mmol/L)	−0.17 (0.04)	<.001	−0.11 (0.04)	.012	.37
	Chol/HDL ratio	−0.20 (0.07)	.008	−0.05 (0.05)	.32	.10
	Ln C-reactive protein (mg/L)	−0.12 (0.08)	.11	−0.11 (0.09)	.24	.92

^a^ *P* values within group.

^b^ *P* value between groups.

^c^BMI=body mass index.

^d^HOMA=homeostatic model assessment.

^e^HDL=high-density lipoprotein.

^f^LDL=low-density lipoprotein (Friedewald).

## Discussion

### Principal Findings

In this study, we describe the effect of a Web-assisted physical activity intervention. We found a significant increase in daily physical activity in the intervention group compared to the control group. Furthermore, we showed that the intervention resulted in a significant improvement in body composition and parameters of glucose metabolism compared to the control group.

To our knowledge, our study is one of the first to show that a Web-based intervention can increase daily physical activity in older people from the general population. Attempts have been made to incorporate the Internet as an additional tool (eg, to generate computer-tailored advice) or intervention strategy (eg, forum, e-buddy) to promote physical activity in the elderly [25,26]. A recently published study showed the effects of a Web-based intervention in seniors, albeit with use of self-reported outcomes [27]. Furthermore, a Web intervention has been used in an elderly study population of 50 patients with chronic obstructive pulmonary disease (COPD) [28].The intervention was primarily directed at COPD self-management, used self-reported exercise as secondary outcome, and compared two different interventions instead of intervention vs a control group, making it difficult to compare their results with our study. Thus far, our study is the first to objectively assess the effects of a Web-based intervention on increasing physical activity in an older population. It is striking that—among an older and inactive population—a Web-based intervention was so effective. The baseline metabolic condition of our participants suggested that we included a study population with indication of the presence of metabolic syndrome: a large proportion of our study sample was overweight or obese, had high waist circumference, and/or used antihypertensive medication. This population, we believe, is particularly relevant for interventions aimed at prevention of age-related cardiovascular and metabolic diseases.

The increase in daily physical activity differed between accelerometer measurement locations; a more pronounced increase was found when using the ankle monitor. At the wrist, the increase in daily physical activity was smaller and not significantly different between groups when looking at total activity counts, but was statistically significant when assessing the increase in validated number of minutes spent in moderate-to-vigorous activity. The larger difference seen between groups in the ankle monitor compared to the wrist monitor is in line with our hypothesis that the ankle location is more sensitive to detect differences in daily physical activity such as cycling behavior. The Dutch population has the highest bicycle use worldwide, and it is likely that especially cycling and walking were stimulated in the inactive study population. In the control group, we also found a small but significant increase in daily physical activity as measured by wrist and ankle accelerometers, but not when assessing the number of minutes spent in moderate-to-vigorous activity. We expected an increase in physical activity in the control group as well, due to an increased awareness of physical activity because of the repeated trial-related measurements [29]. This finding illustrates the need for performing studies in a controlled manner and strengthens our finding that, despite this observation in the controls, the intervention group increased daily physical activity significantly more. Furthermore, there is a need for better understanding the measurements of physical activity patterns from the accelerometer to further underpin the behavior changes associated with such interventions.

Few data exist on the effect of Web interventions directed at physical activity on metabolic health in the general population. Three previous smaller Web-based intervention trials studied outcomes related to metabolic health in populations without chronic disease. Bosak et al studied the effect of a 6-week Web intervention directed at increasing physical activity in 22 participants (mean age 50 years) with metabolic syndrome but found no significant improvements in physical activity, fitness, or lipid levels [30]. After a 16-week Web-based physical activity intervention in inactive adults (mean age 41 years), Carr et al [12] found some improvement in triglycerides levels in the intervention group (n=14) only, but not in any other markers of body composition, lipid, or glucose metabolism, and differences were not compared to the control group (n=18). Hurling et al studied the effects of a 9-week Web-based intervention on anthropometric outcomes and blood pressure in 77 subjects (healthy adults with a mean age of 40 years) [13] and found a significant decrease in body fat between groups, but no effects regarding systolic or diastolic blood pressure. In our study, we also found no significant effect on systolic blood pressure and markers of glucose metabolism. Part of the explanation of why our results differ from the previous studies is that we studied a large sample of older participants with overweight and an inactive lifestyle, likely resulting in a higher burden of metabolic derangement. This has resulted in a higher statistical power to detect differences in health compared to the smaller studies. Alternatively, our intervention may have been especially effective since it was able to deliver personalized feedback on physical activity levels, thereby stimulating behavior change. Finally, we may have selected participants that were more motivated than the participants of the other studies, possibly as the result of chance or our selection process.

### Limitations and Strengths

In our study, we used a waitlist control group. In general, a potential bias in this design is the existence of attention bias, meaning that results could be achieved due to the extra attention given to the intervention group compared to the control group, instead of intervention aspects such as goal setting and self-monitoring. However, the attention given to the intervention group in our study comprised emailing and contact with the coach, which was an essential part of the intervention program under study. Therefore, we believe that the intervention was effective in increasing physical activity and improving metabolic health.

The present study shows the large potential of using Web-assisted interventions for increasing physical activity and increasing metabolic health in a very relevant and aging population. We were able to include over 200 highly motivated participants and improve their metabolic profile within 3 months. However, it is unclear whether compliance can be sustained and whether long-term positive effects can be expected. The very few studies that reported on longer term follow-up and showed a significant increase in PA also after a shorter follow-up period, suggested that physical activity may increase further after 12 compared to 6 months [31]. This study, however, was performed in a primary care setting and used non-Web-based digital intervention methods such as face-to-face counseling. Evidence for long-term effectiveness of Web interventions is therefore required.

Our study population consisted of highly educated and motivated participants, which may hamper the generalizability of our results. Future study should assess the effects of Web-based interventions in elderly in a primary care setting using a population that better represents the general population. A drawback of the present study was that we did not record any dietary behaviors. It is possible that changes in diet account for a proportion of the observed beneficial effects on metabolic health. On one hand, it would be interesting to study which dietary changes are associated with increasing metabolic health, and insight in such behavior could increase the potential to increase effectiveness of such the intervention by specific coaching on this subject. On the other hand, a potential implicit role for dietary factors in the observed effect in the present study does not mitigate the relevance of these results. Another drawback is the fact that we did not have data on longer term follow-up.

The main strength of our study was the use of objectively measured daily physical activity. The majority of studies directed at increasing physical activity used self-reported physical activity measures, which could have resulted in an overestimation of the effect size. More recent studies used pedometers, which are unable to assess all types of physical activity and to give direct feedback to the wearer. With the use of tri-axial accelerometers, outcome assessment was blinded for participants as well as for study physicians and nurses. Another strength of the study was the use of a home-based intervention. This minimized the need for face-to-face contact and subsequently may have explained the low drop-out rate and high adherence to the intervention [6].

### Conclusions

Our results show that using a Web-based intervention in older inactive people at risk for metabolic disease increases daily physical activity and improves metabolic health after 3 months. High retention rates in the intervention group were found, indicating that this Web-based intervention was feasible for use in an older population. Our findings show the large potential of Web-based interventions for large scale prevention of metabolic deregulation in a rapidly aging population.

## Multimedia Appendix 1

Screenshot of DirectLife intervention program and accelerometer.

## Multimedia Appendix 2

Example of DirectLife program and physical activity output.

## Multimedia Appendix 3

Screenshot of a personal plan.

## Multimedia Appendix 4

CONSORT-EHEALTH checklist V1.6.2 [32].

## Abbreviations

BIA: bio-electrical impedance
BMI: body mass index
COPD: chronic obstructive pulmonary disease
GPPAQ: general physician physical activity questionnaire
HbA1c: glycated hemoglobin
HDL: high-density lipoprotein
HOMA: homeostatic model assessment
ITT: intention to treat
LDL: low-density lipoprotein
MET: metabolic equivalentœ
MVPA: moderate-to-vigorous physical activity
PA: physical activity
SE: standard error
