# Supplementary figures and images for: Effects of a Web-Based Intervention on Physical Activity and Metabolism in Older Adults: Randomized Controlled Trial
Source: J Med Internet Res. 2013 Nov 6;15(11):e233. doi: 10.2196/jmir.2843 (PMC3841355; doi:10.2196/jmir.2843)

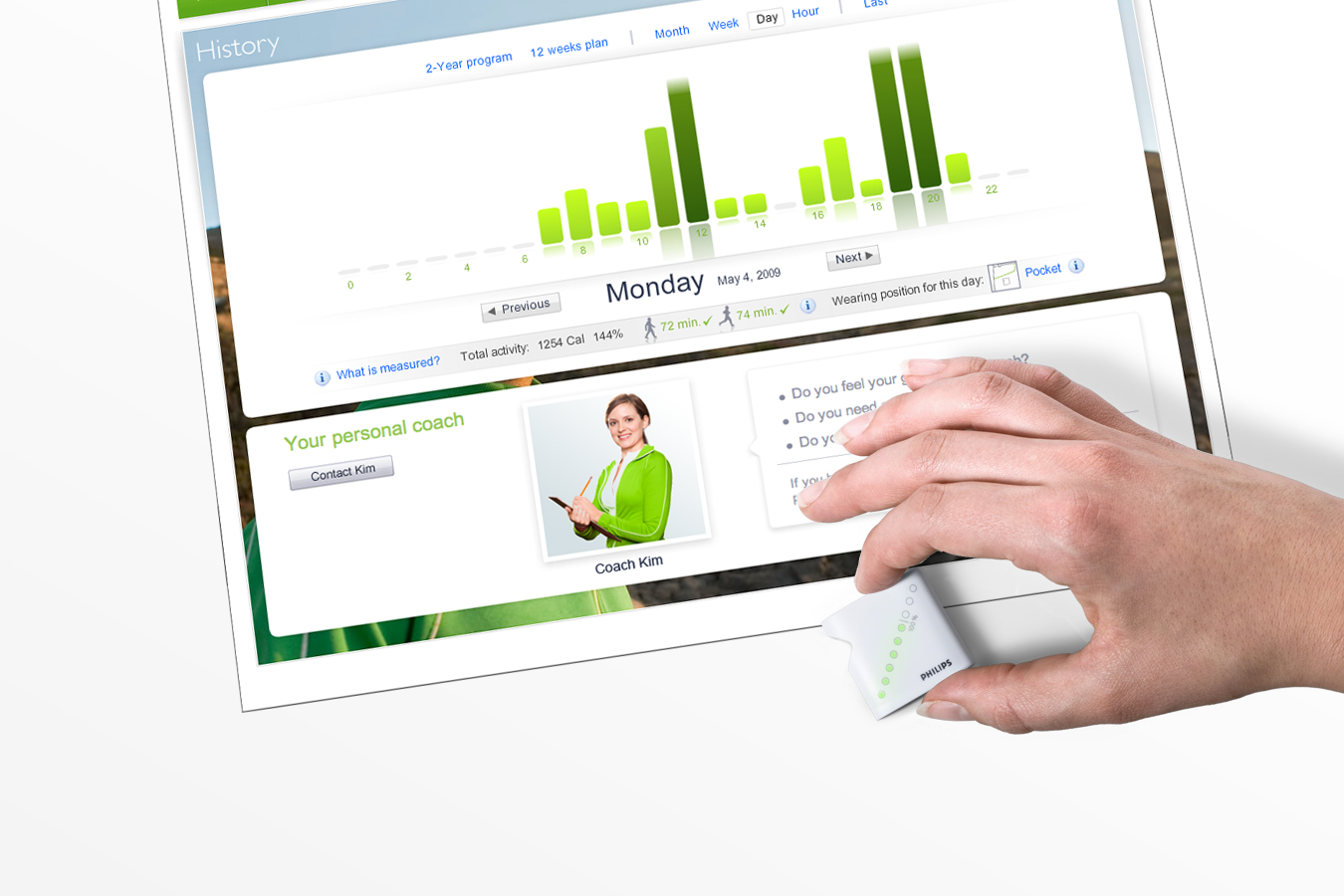

Supplement: Supplementary file 1 [file jmir_v15i11e233_app1.png]

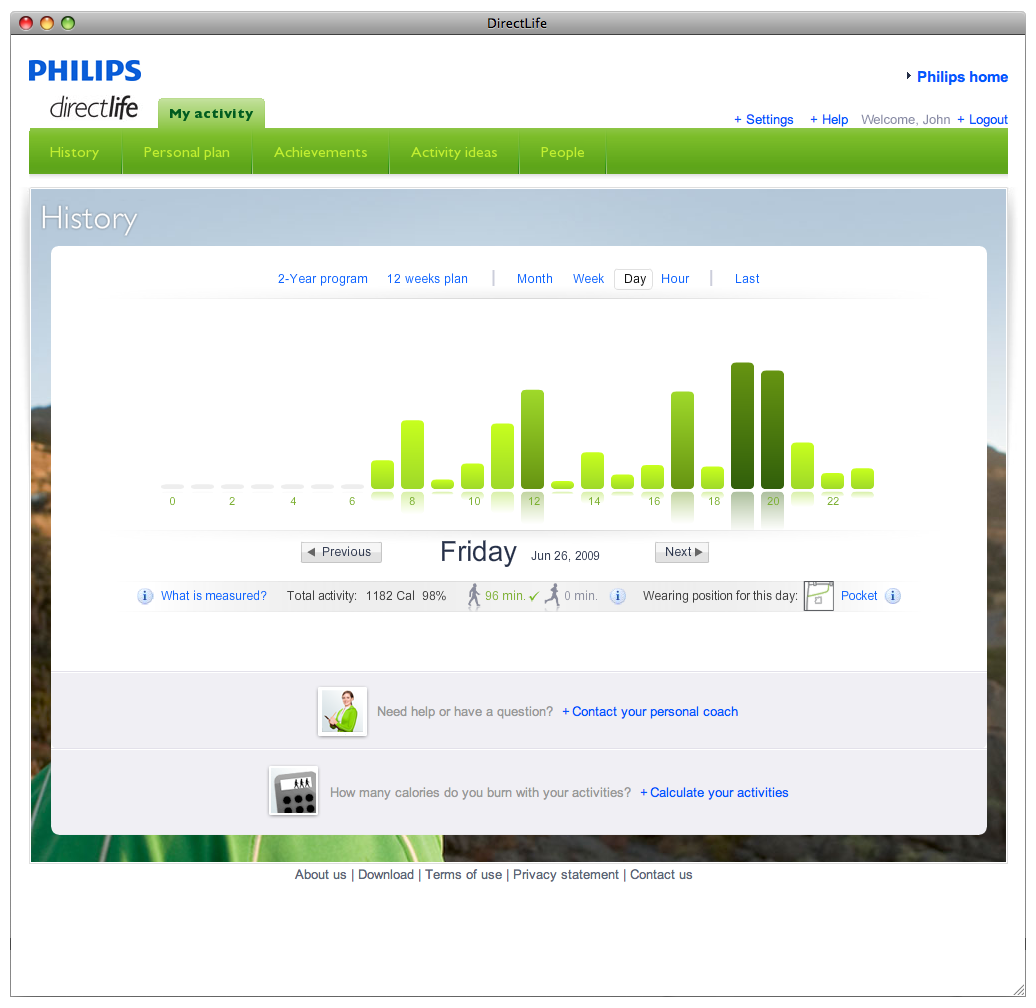

Supplement: Supplementary file 2 [file jmir_v15i11e233_app2.png]

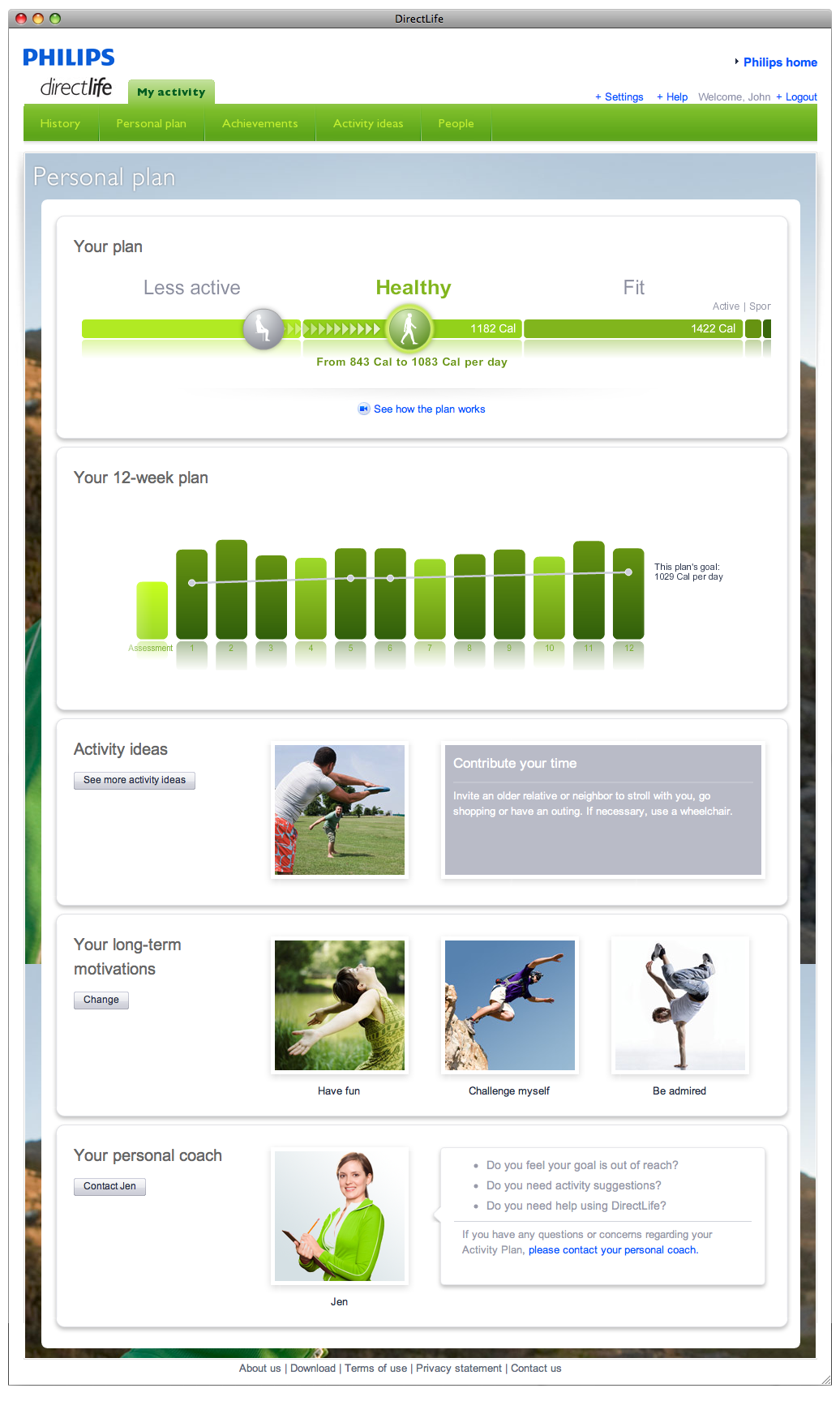

Supplement: Supplementary file 3 [file jmir_v15i11e233_app3.png]
